# Neural Crest Cells Isolated from the Bone Marrow of Transgenic Mice Express JCV T-Antigen

**DOI:** 10.1371/journal.pone.0065947

**Published:** 2013-06-21

**Authors:** Jennifer Gordon, Ilker K. Sariyer, Marisol De La Fuente-Granada, Brian J. Augelli, Jessica Otte, S. Ausim Azizi, Shohreh Amini, Kamel Khalili, Barbara Krynska

**Affiliations:** 1 Department of Neuroscience, Center for Neurovirology, Temple University School of Medicine, Philadelphia, Pennsylvania, United States of America; 2 Department of Neurology, Temple University School of Medicine, Philadelphia, Pennsylvania, United States of America; 3 Shriners Hospitals Pediatric Research Center, Center for Neural Repair and Rehabilitation, Temple University School of Medicine, Philadelphia, Pennsylvania, United States of America; 4 Department of Biology, College of Science and Technology, Temple University, Philadelphia, Pennsylvania, United States of America; University of Medicine and Dentistry of New Jersey, United States of America

## Abstract

JC virus (JCV), a common human polyomavirus, is the etiological agent of the demyelinating disease, progressive multifocal leukoencephalopathy (PML). In addition to its role in PML, studies have demonstrated the transforming ability of the JCV early protein, T-antigen, and its association with some human cancers. JCV infection occurs in childhood and latent virus is thought to be maintained within the bone marrow, which harbors cells of hematopoietic and non-hematopoietic lineages. Here we show that non-hematopoietic mesenchymal stem cells (MSCs) isolated from the bone marrow of JCV T-antigen transgenic mice give rise to JCV T-antigen positive cells when cultured under neural conditions. JCV T-antigen positive cells exhibited neural crest characteristics and demonstrated p75, SOX-10 and nestin positivity. When cultured in conditions typical for mesenchymal cells, a population of T-antigen negative cells, which did not express neural crest markers arose from the MSCs. JCV T-antigen positive cells could be cultured long-term while maintaining their neural crest characteristics. When these cells were induced to differentiate into neural crest derivatives, JCV T-antigen was downregulated in cells differentiating into bone and maintained in glial cells expressing GFAP and S100. We conclude that JCV T-antigen can be stably expressed within a fraction of bone marrow cells differentiating along the neural crest/glial lineage when cultured *in vitro*. These findings identify a cell population within the bone marrow permissible for JCV early gene expression suggesting the possibility that these cells could support persistent viral infection and thus provide clues toward understanding the role of the bone marrow in JCV latency and reactivation. Further, our data provides an excellent experimental model system for studying the cell-type specificity of JCV T-antigen expression, the role of bone marrow-derived stem cells in the pathogenesis of JCV-related diseases and the opportunities for the use of this model in development of therapeutic strategies.

## Introduction

JC virus (JCV) is a ubiquitous human polyomavirus and the etiological agent of the demyelinating disease, progressive multifocal leukoencephalopathy (PML) [1]. PML has been most frequently observed in immunosuppressive states such as in patients with HIV/AIDS or following solid organ transplantation [2]. PML was a rare disease, however, recent reports have shown an increased occurrence of PML related to the treatment of patients with monoclonal antibody therapies such as natalizumab, rituximab and efalizumab [3]. In addition to its role in PML, numerous studies have demonstrated the transforming ability of the JCV early protein, T-antigen, in various experimental models and have shown its association with some human cancers either by the occurrence of the cancer concomitantly with PML or by the molecular detection of viral DNA in neoplastic cells (reviewed by [4], reviewed by [5]).

JCV infection most likely occurs in childhood and, when the immune system is modulated, JCV emerges from latency to become reactivated in the CNS leading to PML [6]. Attempts to understand the latency and dissemination of this virus have suggested that the bone marrow plays an essential role in harboring persistent virus and may serve as a vehicle to circulate the virus throughout different organs of the body. It has been hypothesized that JCV persistently infects bone marrow hematopoietic stem/progenitor cells and that circulating B-cells, which arise from the hematopoietic lineage, most likely transport the virus to the sites of latency or lytic replication within the brain [3]. However, recent studies of PML occurring in multiple sclerosis patients have shown that CD34 positive hematopoietic cells mobilized to the circulation by natalizumab do not contain JCV DNA [7]. Thus, while JCV DNA can be extracted from bone marrow aspirates, the cells of origin in which this virus persists within the bone marrow and the exact role of hematopoietic cells in the pathogenesis of JCV related diseases remain unclear.

Because JC virus persists in the bone marrow [3], a better understanding of the cell types within the bone marrow in which JCV T-antigen can be expressed is important to understanding JCV latency, reactivation and dissemination. Constitutive transgenic mice that harbor the complete coding sequence for JCV T-antigen under the control of the JCV promoter in all cells provide an excellent model to study cell-type specific JCV T-antigen expression. Studies on several lines of transgenic mice which contain the entire gene for JCV T-antigen under the control of its own promoter have shown that JCV exhibits restricted tissue specificity for cells of the central nervous system as well as neuroepithelial cells and neural crest derivatives that give rise to most of the peripheral nervous system [8]–[11]. Recent studies showing the presence of neural cells in earlier unexpected locations such as the bone marrow [12], [13] have prompted us to examine the expression of JCV T-antigen in cells isolated from the bone marrow. We hypothesized that the JCV T-antigen transgene could be expressed in neural cells derived from the bone marrow of JCV T-antigen transgenic mice. To address this question, we isolated bone marrow cells from transgenic mice which contain the entire gene for JCV T-antigen under the control of its own promoter [8] and cultured these cells under conditions which allow for enrichment of the neural subpopulation. Our results show that JCV T-antigen was expressed in bone marrow cells of neural crest lineage suggesting a possible role for these cells in the establishment of JCV latency and viral activation.

## Methods

### JCV T-antigen Transgenic Mice

JCV T-antigen transgenic mice were generated by microinjection of the BalI-NcII fragment of pJC-CY Archetype strain of the JC virus (Genbank AB038249.1) which contains the entire JCV Archetype promoter sequence and the complete early coding region as described previously [8].

### Isolation and Culture of Neural Crest Cells

Primary cultures of MSCs were obtained from femurs and tibias of adult JCV T-antigen transgenic mice ([8]) according to our previously established protocols [14], [15]. All animal studies in this project were performed under protocols approved by Temple University’s Institutional Animal Care and Use Committee (IACUC). In brief, mice were euthanized, femurs and tibias were removed and, using a scalpel blade, bone was opened lengthwise and the bone marrow was washed out under sterile conditions and plated in complete media containing α-MEM (Invitrogen) supplemented with 20% fetal bovine serum (FBS, Atlanta Biologicals), 2 mM L-glutamine, 100 µg/mL penicillin/streptomycin, and 25 ηg/mL amphotericin B (Mediatech).The MSCs were isolated from the hematopoietic component of the bone marrow by their adherence to tissue culture plastic. The non-adherent cells were removed after 48 hours and the adherent cells were thoroughly washed twice with phosphate-buffered saline (PBS) and the media replaced. After an additional four days in culture, cells were harvested with trypsin (0.25%) and cultured in standard mesenchymal media composed of α-MEM media supplemented with 20% FBS or incubated with serum-free Neurobasal media supplemented with 20 ηg/µL of epidermal growth factor (EGF) (Invitrogen) and 20 ηg/µL of basic fibroblast growth factor (bFGF) (Invitrogen) and B27 (1∶50; Invitrogen) for 2–3 weeks. Cells from the initial passage cultured in serum-free Neurobasal media were harvested and expanded for future applications. For passaging, cells were cultured at the density of 5,000 cells per cm^2^ in Neurobasal media supplemented with 20 ηg/µL EGF and 20 ηg/µL of bFGF and B27 (1∶50), subconfluent growing cells were collected by washing with PBS, dissociation with trypsin (0.05%) followed by inactivation of trypsin with media containing serum.

### Immunocytochemistry and Antibodies Utilized

For immunocytochemistry, cells were fixed in 4% paraformaldehyde in PBS for 15 minutes, permeabolized with 0.1% Triton X-100 for 10 min at room temperature and subsequently blocked with 10% goat serum in 1X PBS for one hour at room temperature. For the detection of T-antigen, a mouse monoclonal anti-SV40 T-antigen, which cross-reacts with JCV T-antigen was used (pAb416, DP02, 1∶200 dilution, Calbiochem). Cellular markers were detected with a mouse monoclonal antibody for SOX-10 (MAB2864, 1∶100 dilution, R&D Systems), a rabbit polyclonal antibody for NGR1R p75 (MAB365, 1∶100 dilution, Millipore), and a mouse monoclonal antibody for nestin (611659, 1∶200 dilution, BD Bioscience). Other primary antibodies utilized in this study included a rabbit polyclonal antibody against GFAP (Z0334, 1∶100 dilution, DAKO) and a mouse monoclonal anti-S100 beta protein antibody (M7221, 1∶200 dilution, DAKO). The fixed cells were incubated with primary antibodies at room temperature while shaking for three hours followed by species-specific AlexaFluor 555 secondary antibody (Invitrogen, 1∶1000) for one hour at room temperature in the dark. Controls with secondary antibodies alone were used to determine the background staining. Slides were coverslipped and examined using a Nikon Eclipse E1000 fluorescence microscope using ACT-1 software.

### FACS Analysis

Fluorescent activated cell sorting (FACS) analysis of neural crest cells and MSCs was performed with mouse monoclonal anti-T-antigen antibody. Briefly, cultured cells were harvested, washed in PBS containing 0.1% sodium azide, resuspended in fixation/permeabilization solution (eBioscience), and incubated for 30 min at room temperature. Subsequently, cells were washed with PBS containing 0.1% sodium azide and 2% FBS and stained with anti T-Antigen primary antibody or mouse IgG_2A_ isotype control antibody (MAB0031, R&D Systems) for 40 min. The cells were then washed with PBS containing 0.1% sodium azide and incubated with Alexa Fluor 555 goat anti mouse IgG secondary antibody (Invitrogen) for 30 min at room temperature in the dark. Labeling data was acquired in a FACSCalibur™ flow cytometer (BD Biosciences) and the resulting data were analyzed using Summit software (DAKO).

### Semi-quantitative RT-PCR

Reverse transcriptase-PCR detection of JCV early genes was performed as described before [16], [17]. Briefly, total cellular RNA was extracted from mesenchymal and neural crest cells by using the Qiagen RNeasy kit according to the manufacturer’s recommendations. After treatment with DNase I, followed by phenol/chloroform extraction and ethanol precipitation, cDNAs were synthesized using M-MuLV reverse transcriptase. RNA templates were removed by RNase H digestion. A total of 100 ηg cDNA was used as template for PCR reactions. PCR reactions were amplified between 25–35 cycles to result in PCR amplification within the exponential phase of the PCR curve (Gene Amp 9700, Applied Biosystems). Positive controls consisted of PCR amplifications with JCV Mad-1 genomic DNA and cDNA synthesized from RNA isolated from T98G cells that were transfected with pCDNA3.1-JCV-early plasmid DNA which contains the sequence for the pre-mRNA transcript. PCR amplification with cDNA template obtained from RNA isolated from untransfected T98G cells was used as a negative control. RT-PCR reactions of the JCV-early region splicing were performed using following primers: PF (Mad-1 4801-4780): 5′- CCTGATTTTGGTACATGGAA -3′ and PR (Mad-1 4291–4313): 5′ GTGGGGTAGAGTGTTGGGATCCT -3′. GAPDH was amplified using following primers: GAPDH- Forward: 5′- TTCTCCCCATTCCGTCTTCC -3′, GAPDH-reverse: 5′-GTACATGGTATT CACCACCC-3′. Amplified gene products were resolved on a 2.5% agarose gel and visualized after ethidium bromide staining. To analyze relative expression of different mRNAs, the amount of cDNA was normalized based on signal obtained from the ubiquitously expressed housekeeping gene, GAPDH.

### Differentiation

For osteogenic differentiation, cells were plated at a density of 1×10^4^ cells per cm^2^ and cultured in 10% FBS in DMEM media (Invitrogen) for 5–6 days. Then media was changed to osteogenic induction media containing 10 mM β-glycerolphosphate (Sigma), 0.2 mM ascorbic acid (Sigma) and 10^−8^ M dexamethasone in DMEM containing in 10% FBS and cultures were incubated for three weeks. The media was replaced every 3 to 4 days. To assess mineralization, cells were washed with PBS, fixed in 100% ice-cold methanol for 10 min, and stained for 15 min with 1 mL of Alizarin Red (pH 4.1, Sigma) on a shaker at room temperature. The specimens were then washed three times with dH_2_O and examined under a light microscope. For Schwann cell differentiation, 1×10^4^ cells per cm^2^ were seeded on Matrigel thin-coated (BD Biosciences, manufacturer’s protocol) glass coverslips in DMEM supplemented with N2 (1∶100; Invitrogen) plus the addition of 10 ηg/µL of bFGF (Invitrogen), 10 ηg/µL neuregulin (R&D Systems) and 5 µM forskolin (Sigma) for a week with 50% media refreshed every 2–3 days. Cultures were then fixed in 4% paraformaldehyde and examined by immunocytochemistry.

## Results

### Isolation and Culture of Neural Cells from the Bone Marrow

**Figure 1:**
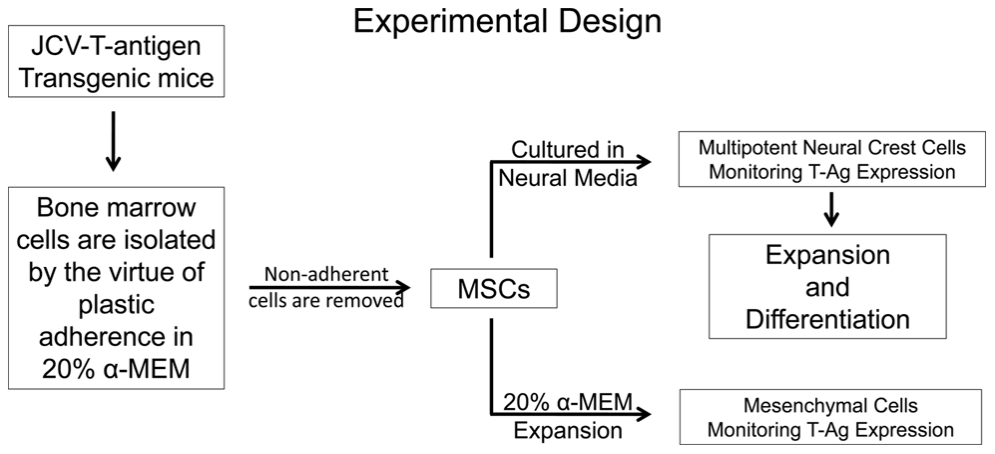
Culturing of bone marrow cells isolated from adult JCV T-antigen transgenic mice. Mesenchymal stem cells (MSCs) were isolated from the bone marrow of adult JCV T-antigen transgenic mice by the virtue of plastic adherence when cultured in mesenchymal media composed of α-MEM containing 20% fetal bovine serum. Non-adherent cells were removed and discarded while adherent cells were then transferred to neural stem cell media (containing bFGF and EGF) for 2–3 weeks or maintained in mesenchymal media. Cells in both culture conditions were monitored for growth and analyzed for expression of JCV T-antigen. Neural crest cells were further expanded and analyzed for expression of neural crest markers and differentiation into glial and osteogenic components to confirm their neural crest origin.

**Figure 2:**
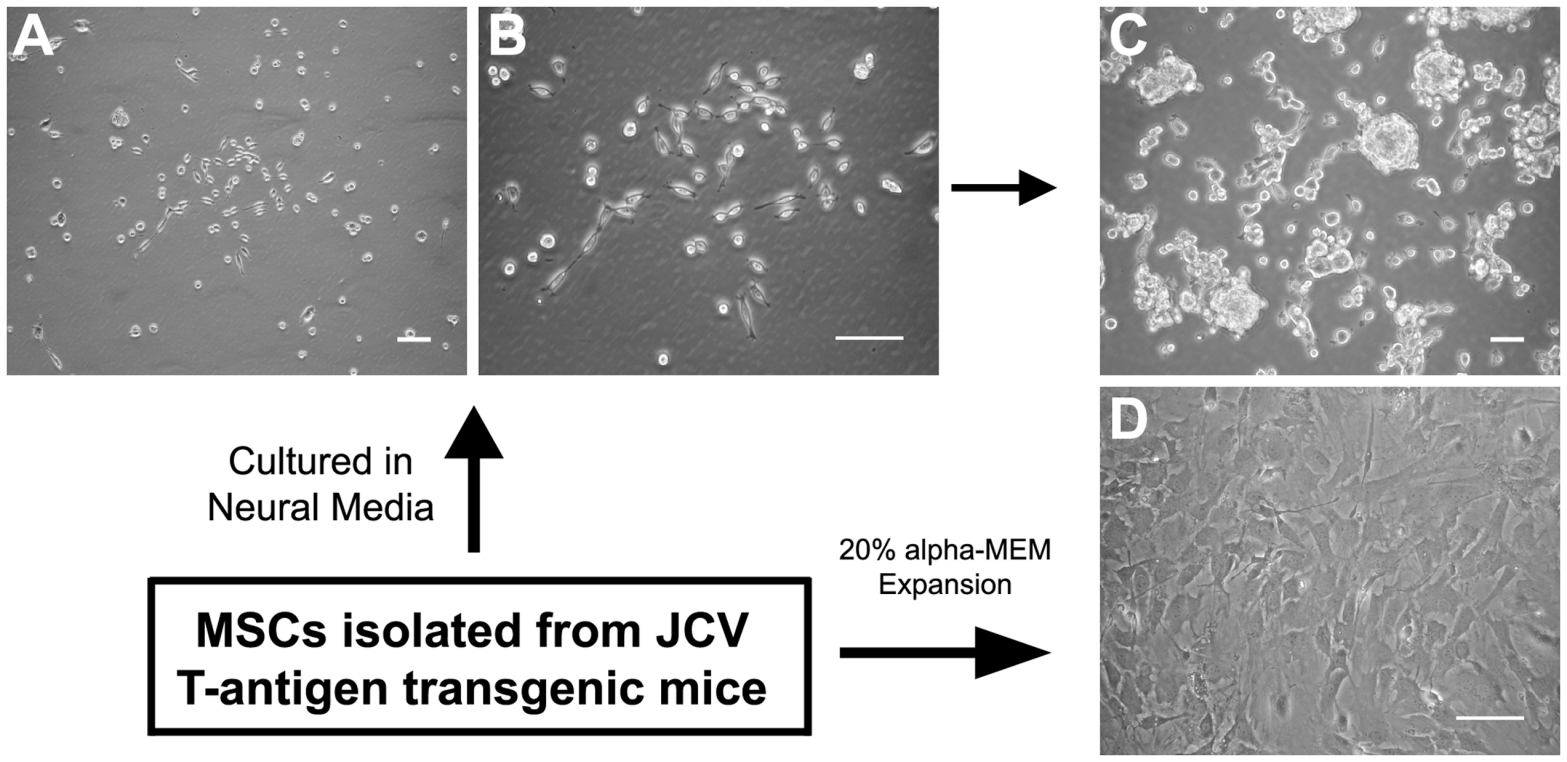
Culture characteristics of MSCs isolated from the bone marrow of JCV T-antigen transgenic mice. Phase contrast micrographs of MSCs cultured in neural stem cell media demonstrate small proliferating cells observed after 2–3 weeks in culture (A and B), which formed semi-attached spheres after cells reached confluency (C). MSCs cultured in α-MEM with 20% FBS formed a densely packed monolayer and exhibited morphology typical of mesenchymal cells (D). (Phase contrast, Scale bar: 100 µm.).

Given the rarity of cells of neuroepithelial origin in bone marrow aspirates and the possibility of their *in vitro* enrichment in cultures of mesenchymal stem cells (MSCs), we first isolated the MSC fraction of the bone marrow from JCV T-antigen transgenic mice by the virtue of their adherence to tissue culture plastic in α-MEM media supplemented with 20% fetal bovine serum which supports the growth of mesenchymal cells. At the first passage, MSCs isolated from the bone marrow of JCV T-antigen transgenic mice were subcultured and maintained in serum-free neural stem cell media supplemented with bFGF and EGF or in α-MEM supplemented with 20% fetal bovine serum. Cells grown under both conditions were monitored for growth and analyzed for the expression of JCV T-antigen (Fig. 1). After being cultured for 2–3 weeks in serum-free media in the presence of bFGF and EGF, small proliferating bipolar cells were observed in the cultures (Fig. 2A, B). Cultured cells gradually detached from the plastic tissue culture dish and aggregated forming semi-attached spheres as the cultures proliferated (Fig. 2C). Cells cultivated in standard mesenchymal cell culture conditions in the presence of serum were flat, strongly adherent to tissue culture plastic, and displayed contact inhibition and a morphology typical of stromal cells (Fig. 2D). We followed the growth of these cells and characterized their expression of neural markers and JCV T-antigen.

### Characterization of Cell Lineage and T-antigen Expression

**Figure 3:**
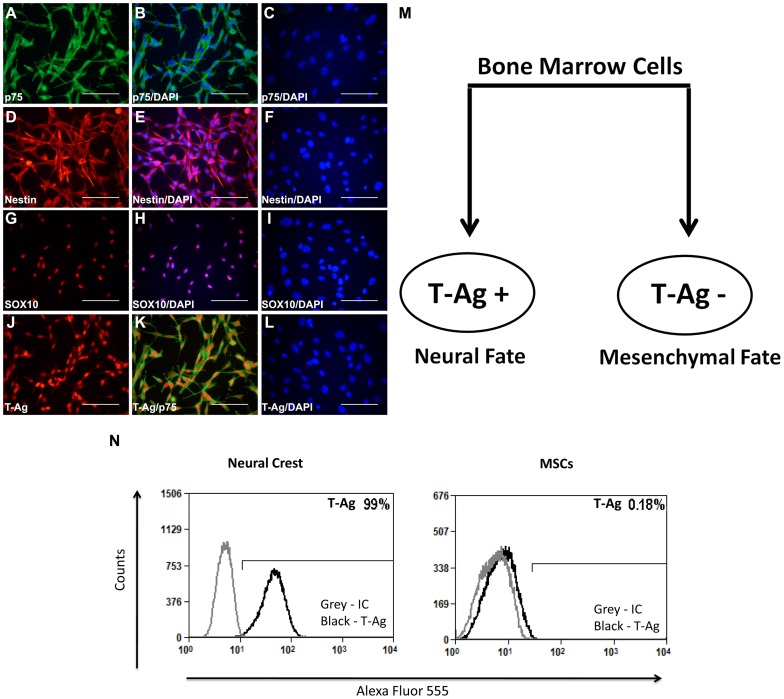
JCV T-antigen is expressed in neural crest cells. Fluorescent images of bone marrow cells isolated from JCV T-antigen transgenic mice cultured in neural conditions show that these cells are positive for p75 (A,B), nestin (D,E) and SOX-10 (G,H), which are all markers of the neural crest. JCV T-antigen (J,K) was detected in the nucleus of p75 positive cells. Bone marrow cells cultured in mesenchymal media were negative for p75, nestin and SOX-10, and did not express JCV T-antigen (C, F, I, L). Cell nuclei were visualized with DAPI. (Scale bar: 100 µm). (M) Diagram illustrating expression of JCV T-antigen in bone marrow cells. T-antigen expression was associated with neural crest lineage cells of the bone marrow cultured in serum-free neural stem cell media supplemented with bFGF and EFG (left panel), while mesenchymal cells maintained in α-MEM in the presence of 20% serum were negative for T-antigen (right panel). (N) FACS analysis of neural crest cells and MSCs demonstrates positivity for JCV T-antigen in 99% of neural crest cells and lack of JCV T-antigen expression in MSCs.

**Figure 4:**
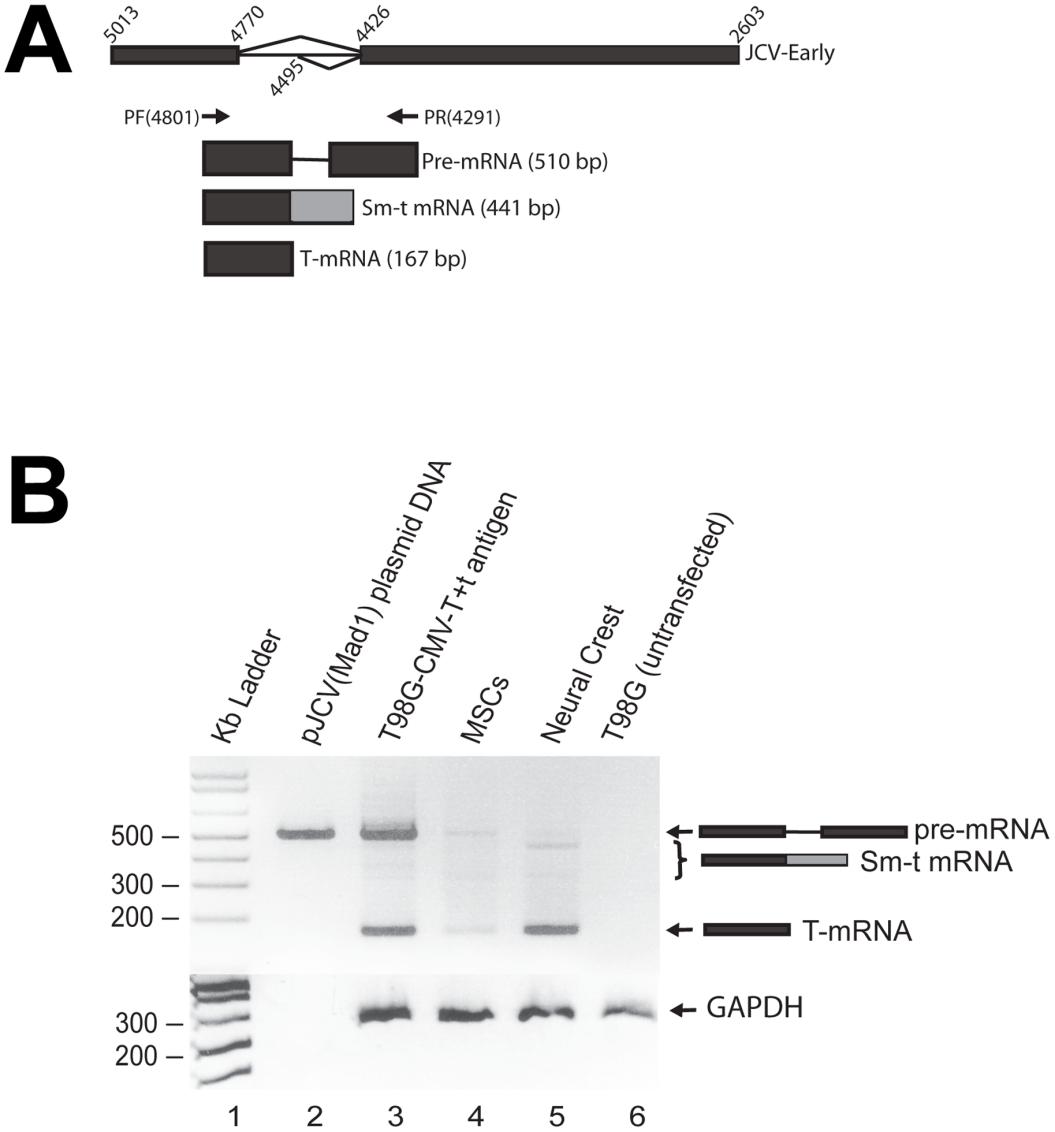
Detection of JCV T-Ag mRNAs in neural crest cells by RT-PCR. **A.** Schematic representation of JCV early region unspliced and spliced RNAs and the size of the amplification products with a primer set (PF and PR), used for the amplification of JCV gene products in panel B. **B.** RT-PCR analysis of JCV pre-mRNA, and the large and small T-antigen alternatively spliced early transcripts in RNA isolated from JCV T-antigen transgenic mouse MSCs and neural crest cells. Weak signal of large T-antigen mRNA and little or no detection of small t-antigen transcript in MSCs and up-regulation of large T-antigen and small t-antigen transcripts in neural crest cells. Lane 1: Kb ladder, lane 2: JCV Mad-1 genomic plasmid control DNA, lane 3: RNA isolated from T98G cells transfected with pCDNA3.1-JCV-early plasmid DNA (positive control), lane 4: RNA isolated from MSCs, lane 5: RNA isolated from neural crest cells, lane: 6 RNA isolated from untransfected T98G cells (negative control). GAPDH amplified from the same cDNA samples was used as input control.

To characterize the cultured cells, we performed immunocytochemical analysis and found that all cells cultured in serum-free media with the addition of bFGF and EGF expressed strong p75 immunoreactivity, indicating a neural crest lineage (Fig. 3 A,B). In addition, all cultured cells expressed two additional neural crest markers, nestin (Fig. 3 D,E) and SOX-10 (Fig. 3 G,H) [18]–[20]. Immunocytochemical analysis of T-antigen expression revealed the presence of nuclear expression of the transgene in all neural crest cells, indicating that the JCV T-antigen promoter is active and T-antigen is expressed in bone marrow-derived cells of neural crest lineage (Fig. 3 J, K). In contrast, plastic adherent cells cultured under standard mesenchymal cell culture conditions in the presence of serum were negative for expression of T-antigen and did not express neural crest markers (Fig. 3 C, F, I, L) indicating that expression of T-antigen is associated with neural fate of bone marrow cells (Fig. 3 M) To complete the characterization of JCV T-antigen expression, we performed fluorescence activated cell sorting (FACS) analysis of both the neural crest and mesenchymal cell cultures. FACS analysis with anti-T-antigen antibody confirmed that 99% of the neural crest cells were positive for JCV T-antigen while JCV T-antigen expression was absent in the mesenchymal cells (Fig. 3 N). In support of this finding, reverse transcriptase-polymerase chain reaction (RT-PCR) analysis of RNA was performed to detect the JCV early transcript, which encodes large and small T-antigens in a single alternatively spliced transcript. Primers designed to detect the pre-mRNA, or to distinguish between the spliced transcripts for the large versus the small T-antigens revealed RNA transcripts encoding the JCV-early genes (large T-antigen and small t-antigen) in RNA extracted from neural crest cells, while; a weak signal for RNA encoding large T-antigen and little or no message of the small t-antigen transcript was observed in RNA extracted from mesenchymal cells (Fig. 4 A, B). The MSC-derived neural crest lineage cells that expressed JCV T-antigen could stably proliferate in long-term cultures, while the MSCs cultured in mesenchymal cell culture conditions, were negative for T-antigen, were only capable of limited proliferation and failed to expand in long-term cultures. Thus, we conclude that the JCV T-antigen transgene is expressed in neural crest lineage cells of the bone marrow isolated from JCV T-antigen transgenic mice.

### Differentiation of Neural Crest Cells into Neural and Non-neural Cells

**Figure 5:**
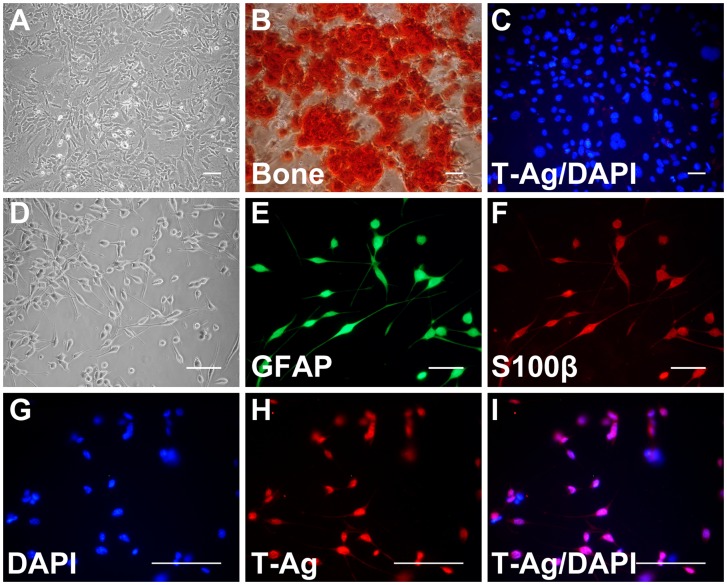
Induction of JCV T-antigen positive neural crest cells to differentiation into neural and non-neural cells. Phase contrast micrograph (A) and Alizarin red staining (B) of JCV T-antigen positive neural crest cells after differentiation into bone and los expression of JCV T-antigen (C). Phase contrast view (D) and fluorescent images demonstrating JCV T-antigen positive neural crest lineage cells after differentiation into Schwann-like cells positive for GFAP (E) and S100 (F). Schwann-like cells expressed JCV T-antigen (G-I). Cell nuclei were visualized with DAPI. (Scale bar: 100 µm.).

Neural crest cells can give rise to a wide variety of cell types that are developmentally derived from the neural crest [21]. To determine whether the bone marrow-derived neural crest cells expressing T-antigen maintained their ability to differentiate and investigate the capacity of the JC viral promoter to express T-antigen in these cells, we induced their differentiation into neural and non-neural cell types and analyzed expression of T-antigen in these cells in parallel. Following priming of the neural crest cells in serum, we induced their differentiation into osteocytes by culturing in osteogenic media. In response to osteogenic inductive conditions, these cells formed mineralized nodules and demonstrated staining with Alizarin red (Fig. 5 A, B). Immunostaining for T-antigen expression rendered negative results in osteogenic cultures, indicating that the cells acquired an osteogenic phenotype while T-antigen expression was concomitantly down-regulated in differentiating cells (Fig. 5 C). Subsequently, JCV T-antigen expressing neural crest cells cultured in serum-free neural stem cell media supplemented with bFGF and EGF were transferred to adherent conditions and allowed to differentiate into glia. Following adherence and outgrowth for 6–8 days, the plated cells assumed Schwann cell –like morphology and were strongly positive for the astrocytic marker, GFAP, as illustrated in Figure 5 D, E. Further, immunocytochemistry showed that these cells also expressed S100 beta (Fig. 5 F). Immunostaining for T-antigen expression revealed positive staining in these cells (Fig. 5 G–I). Thus, Schwann cell-like cells maintained expression of JCV T-antigen during differentiation into glia further supporting expression of JCV T-antigen in glial cells derived from the bone marrow. These findings are in line with previous studies detecting the expression of T-antigen in glial cells and tumors of glial origin in the CNS and PNS [4], [11].

## Discussion

In this study, we have used adult JCV T-antigen transgenic mice to investigate the capacity of the virus to express T-antigen in bone marrow-derived cells. We have shown that JCV T-antigen expression can be detected in bone marrow cells when MSCs were cultured in serum-free media in the presence of bFGF and EGF, i.e. growth factors commonly used for the culturing of neural stem cells. Investigation of their phenotype showed that these proliferating cells expressing JCV T-antigen were positive for p75, SOX10 and nestin, indicating the neural crest characteristics of these cells. A comparison of neural crest cells and mesenchymal cells cultured in the presence of 20% serum in α-MEM media showed that T-antigen expression was associated with the neural crest lineage cells of the bone marrow, rather than mesenchymal cells. When neural crest cells were induced to differentiate into neural crest derivatives, JCV T-antigen expression was downregulated in cells differentiating into bone while glial cells expressing GFAP and S100 remained positive for T-antigen. These findings support previous studies demonstrating the preferential expression of T-antigen in neural cells and, as viral early gene expression is a critical early step in the virus life cycle, suggest that this fraction of neural cells obtained from the bone marrow would be candidates cells in which JCV could potentially establish an infection.

Although JCV is considered a neurotropic virus due to its association with PML, the bone marrow has been shown to be a potential reservoir for JC virus by several groups [22], [23]. The detection of viral DNA in the absence of progeny virus suggests that bone marrow cells may harbor latent virus. However, the cells of origin within the bone marrow and the specificity of the virus for subpopulations of bone marrow cells remain unclear. We isolated MSCs from the bone marrow of JCV T-antigen transgenic mice and showed that T-antigen positive cells arose in cultures of MSCs exposed to neural conditions. T-antigen positive cells, as observed in our cultures, had characteristics of the neural crest and could be neuroepithelial-derived bone marrow cells. The activation of T-antigen expression in neural crest cells after the exposure of bone marrow cells to neural conditions may be indicative of the differentiation of these cells into T-antigen positive neural crest cells in response to a neural environment. Thus, the important observations are the lack of T-antigen expression in MSCs and activation of T-antigen expression in cells isolated under neural culture conditions to yield neural crest cells, suggesting a specific compartment within the bone marrow which may latently harbor JCV and allow reactivation of the virus under permissive conditions. Moreover, given that cells derived from the neural crest populate virtually all tissues of the vertebrate body, our findings suggest that the neural crest may play a role in dissemination of the virus.

To characterize the tissue specific expression of JCV, and to investigate the potential of JCV T-antigen in inducing disease, a number of lines of transgenic mice harboring JCV T-antigen under the control of the JCV promoter have been generated. Some of these mice developed adrenal neuroblastomas [9]. Other lines of mice developed solid mesenteric tumors with tumor cells exhibiting characteristics of primitive epithelial/neuroectodermal origin [24], or solid masses histologically compatible with malignant peripheral nerve sheath tumors [11]. These T-antigen induced tumors might thus arise from a neuroepithelial/neural crest cell type. Recently, we reported that bone marrow-derived MSCs which were transformed by JCV T-antigen in culture led to heterogeneous xenograft tumors with mesenchymal, neural, and neural crest characteristics, consistent with a propensity to become a T-antigen positive neural crest cell [25]. Furthermore, our experiments showing the expression of T-antigen in neural crest cells are consistent with previous studies showing the detection of JCV T-antigen in cells with neural crest characteristics isolated from various tissues of the transgenic mice containing the JCV early region that suffered from dysmyelination [10]. In addition, these studies showed that mesenchymal fibroblasts isolated from JCV-transgenic mice did not express T-antigen [10], which further supports our observation that MSCs cultured under mesenchymal conditions selected for a population of T-antigen negative cells.

JCV is the causative agent of PML in humans and JCV tumorigenesis is well-established in experimental animal models (reviewed by [4], reviewed by [5]). Efforts to understand the latency of this virus have suggested that the bone marrow plays an essential role in harboring and disseminating the virus throughout different organs. Given that the bone marrow has been shown to be a reservoir for JC virus [23] and the richness of cell varieties within the bone marrow [26]–[31], it remains to be investigated whether human cells of neuroepithelial origin harboring the virus can be isolated from bone marrow aspirates in cases of PML occurring in multiple sclerosis patients or other conditions associated with the reactivation of JC virus. Because bone marrow cells are relatively easily accessible; the possibility to obtain human bone marrow-derived stem cells and differentiation of these cells into various lineages would offer the possibility of using these cells as a unique model system for studying the role of bone marrow-derived stem cells in JCV latency, reactivation and the pathogenesis of JCV-related diseases. This model system may also be uniquely suited for the development and validation of novel therapeutic strategies.
